# Fear of the COVID-19 vaccine in a public healthcare system and university setting

**DOI:** 10.1371/journal.pone.0304000

**Published:** 2024-06-25

**Authors:** Roberta de Oliveira Botelho, Carolina Cramer Filgueiras Coelho, Eric Francelino Andrade, Paula Midori Castelo, Vanessa Pardi, Ramiro Mendonça Murata, Luciano José Pereira

**Affiliations:** 1 Department of Medicine, Universidade Federal de Lavras (UFLA), Lavras, MG, Brazil; 2 Department of Pharmaceutical Sciences, Universidade Federal de São Paulo (UNIFESP), Diadema, SP, Brazil; 3 Department of Foundational Sciences, School of Dental Medicine, East Carolina University (ECU), Greenville, NC, United States of America; UV: Universiteti Ismail Qemali Vlore, ALBANIA

## Abstract

Despite the known benefits, some individuals remain apprehensive about receiving the COVID-19 vaccine, which hampers vaccination efforts and the achievement of herd immunity. Therefore, this cross-sectional study aimed to assess vaccination rates and identify factors influencing fear of the COVID-19 vaccine among individuals served by the public healthcare system (Family Health Strategy ‐ FHS) and in a university community in Minas Gerais, Brazil. Surveys were conducted face-to-face with FHS participants and online with university members, employing a free sharing approach on social media. A total of 1896 and 312 responses were collected, respectively. The survey covered sociodemographic information, COVID-19 fear levels, and vaccination status for both individuals and their children Vaccination coverage was 83% among FHS participants and 99.1% in the university setting. Female respondents in both groups exhibited higher levels of COVID-19 fear (p<0.05), with FHS-assisted women reporting greater apprehension towards vaccination (p<0.05). Educated parents demonstrated better understanding of the importance of child vaccination, while younger parents expressed heightened concerns about vaccine side effects. Among FHS participants, women exhibited a 1.6 times higher fear of vaccination compared to men. Additionally, fear of vaccination increased by 1.10 times for each additional point on the COVID-19 Fear Scale (physiological domain). Effective communication strategies and dispelling misconceptions surrounding immunization could alleviate fear and promote vaccination acceptance.

## Introduction

The coronavirus disease 2019 (COVID-19) pandemic, declared by the World Health Organization (WHO) on March 11, 2020, was the subject of numerous studies aimed at the development of safe and effective vaccines against the virus [1]. As a result, due to the global use of immunization, after March 5, 2023, COVID-19 lost its status as a public health emergency of international concern [2]. However, its dissemination and potential for the emergence of new variants remain a global concern in the field of health [3].

Effective control of the COVID-19 pandemic depended on adequate vaccination coverage. In Brazil, four main vaccines were and continue to be used for immunization of the population: Comirnaty (Pfizer/Wyeth), CoronaVac (Sinovac and Butantan), Oxford/Covishield (AstraZeneca and Fiocruz) and the Janssen vaccine (Janssen-Cilag) [4]. However, despite the proven benefits of the available vaccines, vaccine hesitancy has persisted, impairing vaccination coverage and the establishment of herd immunity [5–7].

Vaccine hesitancy is characterized by refusal or delay in accepting vaccination, even when health services are available. This behavior can be influenced by complex factors such as personal, religious, cultural, and political beliefs and issues related to the safety and efficacy of immunization agents [1, 8–11]. The National Immunization Program (PNI) in Brazil, linked to the Unified Health System (SUS), has played a key role in promoting vaccination against COVID-19, especially among the most vulnerable Brazilians [7].

In addition to socioeconomic vulnerability, a low education level has been associated with vaccine hesitancy due to a lack of knowledge about the importance and safety of vaccines [12]. Studies report that health information and knowledge are directly linked to more favorable attitudes toward vaccination [13]. Understanding the reasons for vaccine refusal is crucial for the development of public policies aimed at increasing vaccination coverage [14]. Considering the diversity of Brazil and the multiple aspects involved in vaccine hesitancy, assessing behaviors in primary care gateway settings and in a university setting can help in the development of public health strategies to reduce the number of unimmunized people. Studying both individuals assisted by the public healthcare system and members of the university community is crucial for capturing a wide range of socioeconomic backgrounds and educational levels. By incorporating these distinct populations, diverse perceptions and predictive factors of COVID-19 vaccine fear can be assessed, taking into account the potential impact of socioeconomic vulnerability and higher education on vaccination attitudes and behaviors.

In light of these issues, our study aims to investigate the immunization rate and factors associated with fear of the COVID-19 vaccine in two distinct populations: a public and federal university community and the general population of a city in the south of the state of Minas Gerais, Brazil, in the southeastern region of the country.

## Material and methods

### Ethical considerations

This study received approval from the Ethics Committee for Research Involving Human Subjects at the Federal University of Lavras (UFLA) (CAAE 54996322.1.0000.5148). Participants in the online survey provided written, informed, and voluntary consent by affirmatively indicating their agreement to participate in the research on a specific question through the electronic form. Participants in the in-person survey formally signed the written informed consent form (ICF). It is noteworthy that the survey sample excluded minors, defined as individuals below the age of 18, in compliance with ethical considerations.

### Population and sampling

The city of Lavras is in the interior of the Brazilian state of Minas Gerais, belonging to the Campo das Vertentes mesoregion and the Intermediate Geographic Region of Varginha, in the microregion of Lavras. Lavras is located at a latitude of 21° 14’ 43’’ to the south and a longitude of 44° 59’ 59’’ to the west. In the last demographic census, conducted in 2010, its population was 92,200. In 2021, the estimated population of the city was 105,756 [15]. Lavras has 17 family health units and 2 basic health units [16].

The Federal University of Lavras (UFLA) is also located in the city and is an institution founded in 1908 that offers 38 modalities of on-site higher education courses, 41 master’s programs and 23 doctoral programs in different areas of knowledge, in addition to 3 distance learning courses. At the time of data collection, the institution had 11,100 undergraduate students, 1,850 graduate students, and 1,340 staff members [17].

### Data collection and questionnaires

Data collection for the community assisted by the Family Health Strategy (FHS) was performed both during pre-consultations at health care facilities and through home visits. In the university environment, an online platform was used (Google Forms, Alphabet, Mountain View, CA, USA). The questionnaires were distributed via social networks (Facebook®, Instagram®, and WhatsApp®) and email. A snowball survey strategy was employed to maximize respondent recruitment [18]. This cross-sectional study included only adults over 18 years of age of both sexes who lived in the city in which this study was conducted or who were affiliated with the higher education institution.

The questionnaires used contained questions regarding sociodemographic data such as age, sex, race, occupation, education and average family income. Information was also collected regarding the health history of repsondents, with questions about chronic diseases and continuous medication use. Additionally, participants answered questions related to COVID-19, such as those on confirmed previous infections, vaccination, and perceived risk and fear of contracting the disease. Fear of COVID-19 was quantified using the Brazilian version of the COVID-19 Fear Scale [19], adapted from the original scale developed by Ahorsu et al. [20] This scale comprises seven questions rated on a five-point Likert scale. The score for each domain, as well as the total score, is calculated by summing the points. Consequently, a higher score indicates a higher level of fear of COVID-19. In addition, the participants were asked if they had children under 18 years of age, and if so, questions about their vaccination status were presented [21].

In the population assisted by the FHS, the questionnaires were distirbuted between 06/28/2022 and 09/17/2022. Among university students, data collection occurred between 06/21/2022 and 08/23/2022. In Brazil, as of 07/12/2023, 515,636,659 doses of vaccines against COVID-19 have been administered. Of these, 166,837,435 were second doses of the monovalent vaccine, and 5,053,174 were single doses. The state of Minas Gerais administered 52,499,266 total doses, of which 17,003,267 were second doses and 526,543 were single doses. In the city of Lavras, to date, the numbers are 281,646 total doses, 90,230 second doses and 1,638 single doses [22].

### Statistical analysis

Statistical analysis was performed using SPSS 28.0 software. An alpha level of 5% was adopted, and the analyses were performed separately for data collected remotely from the university community (online survey) and data collected through questionnaires in health units (in-person survey).

The descriptive analysis consisted of means, standard deviations, medians, percentages, and graph analyses. The comparison between continuous and categorical variables was performed using one-way ANOVA and chi-square tests, respectively. The analysis of the differences between the sexes regarding the questions on the fear of COVID-19 was performed using analysis of covariance (ANCOVA) adjusted for age.

For data collected remotely from the university community (online survey), cluster analysis (K-means clustering) was performed to identify groups of participants with similar variables related to sociodemographic and clinical aspects and to fear of receiving the COVID-19 vaccine. The final number of clusters was based on the interpretability and reliability of the clustering method; the differences between the clusters were described by the F test for validation and interpretation purposes.

Regarding the data collected through the application of questionnaires to FHS participants, ordinal logistic regression was used to predict the degree of fear of being vaccinated against COVID-19 (none, low, moderate and very afraid) according to the predictive variables in this study: age, schooling, sex, pregnancy, COVID-19 Fear Scale score, vaccination status, death of a friend/family member as a result of COVID-19, previous COVID-19 infection, chronic disease and whether they were a health worker. The results of the omnibus test and Pearson’s chi-square test were examined to assess the goodness of fit.

Finally, to understand the motivations and fears regarding the vaccination of respondents’ children, four binomial logistic models were adjusted for the following questions: ’Vaccinating my child is important for the health of other people in my family/community’; ’The new vaccines against COVID-19 have more risks than other vaccines (for example, flu vaccines)’; ’Vaccinating my child is a good protective measure’; and ’I am concerned that my child will develop an adverse effect related to the COVID-19 vaccine’, with dichotomous responses (yes/no). The predictive variables sex, death of a friend/family member due to COVID-19, presence of chronic disease, previous COVID-19 infection and health worker status were included in the initial model, and stepwise backward elimination was used to generate the final model. The results of the omnibus test (p<0.05), the Hosmer–Lemeshow test (p>0.05) and Nagelkerke’s R2 were considered to evaluate the quality of the models.

## Results

### Online survey

The description of the sample of participants from the university community who completed the questionnaire in a virtual environment is presented in Table 1. Among the 312 respondents, 134 reported being employees (professors, technicians, or collaborators), and 178 reported being students (150 undergraduate and 28 graduate students). Only one respondent was pregnant, and 81 participants reported having a child under 18 years of age; 99% of the respondents were residents of an urban area.

**Table 1:** Sociodemographic and clinical characteristics of participants in the university community: Online survey.

		Women (n = 158)	Men (n = 154)
**Age** [Mean (SD)]	Years	31.5 (11.2)	35.8 (13.6)
**Skin color** (%)	White	66.5	73.4
	Black	5	4.5
	Brown/Yellow	26	18.8
	Not reported	2.5	3.3
**Education level** (%)	High school	0.7	1.3
	Graduation	54.4	42.2
	Graduate	44.9	56.5
**Family income** (%)	<0,5–1,5 wages/person	24.7*	13.5*
	1,5–3 wages/person	24.0	16.9
	3–6 wages/person	24.0	20.8
	>6 wages/person	18.4*	36.4*
	Not reported	8.9	12.4
**Occupation** (%)	Student	60.8	53.3
	Professor	25.2*	39.6*
	Technical service/employee	14*	7.1*
**Health worker** (%)	yes	15.8	13
**Chronic disease** (%)	yes	22.2	25.3

*p<0.05 (chi-square test)

The distribution of respondents according to sex was quite homogeneous (50.6% women), with a similar distribution between sexes in terms of age, race and health care worker status (p>0.05). There was a difference in the distribution between men and women regarding income and occupation (p<0.05). However, in terms of family income, the distribution was homogeneous (approximately 20% for each group; <0.5–1.5; 1.5–3 and 3–6 wages per family member). In addition, 22% of women and 25% of men reported having some chronic disease, the most cited being obesity (2.6%), diabetes (4.2%) and hypertension (8%) (Table 1).

Table 2 shows the comparison of the variables related to COVID-19 between the sexes. The mean scores for the Fear of COVID-19 scale were 14.3 (±4.6) for women and 13.1 (±4.6) for men. Approximately 50% of the participants indicated a prior history of COVID-19 infection, while over 60% expressed apprehension about contracting the virus. Additionally, a significant portion reported experiencing the loss of a family member or acquaintance due to complications from severe acute respiratory syndrome coronavirus 2 (SARS-CoV-2) infection. The perceived risk of contracting the virus was higher among women (p = 0.044). Regarding the questions on the COVID-19 Fear Scale, women scored higher for the items “I feel uncomfortable thinking about COVID-19” (p<0.001), “When watching news and stories about COVID-19 I become nervous or anxious” (p<0.001) and for the total score of the questionnaire (p = 0.022).

**Table 2:** Description of university community participants according to variables related to COVID-19: Online survey.

		Women (n = 158)	Men (n = 154)
**Already contracted COVID-19** (%)	yes	52.5	48.1
**Family member/friend/acquaintance died of COVID-19**	yes	63.9	62.3
**Fear of contracting COVID-19**	yes	61.4	51.9
**Perceived risk of contracting COVID-19**	low	15.8 ‡	26.7 ‡
	moderate	58.2	54.5
	high	26 ‡	18.8 ‡
**Fear of being vaccinated against COVID-19** (%)	none	79.7	83.7
	low fear	13.9	10.5
	moderate fear	3.8	4.6
	very afraid	2.5	1.3
**Fear of COVID-19 Scale†** [Mean (SD)]	I am more scared …	2.2 (1.0)	2.3 (1.0)
	I feel uncomfortable thinking …	2.7* (1.1)	2.3* (1.2)
	My hands are wet …	1.3 (0.6)	1.3 (0.5)
	I am afraid of losing my life …	2.6 (1.3)	2.4 (1.3)
	When watching the news … I become nervous or anxious	2.7* (1.2)	2.2* (1.2)
	I cannot sleep …	1.3 (0.6)	1.3 (0.6)
	My heart races or flutters …	1.5 (0.9)	1.4 (0.8)
	Total score	14.3* (4.6)	13.1* (4.6)

‡ p<0.05 (chi-square test)

* p<0.05 (ANCOVA test with adjustment for age)

† Cronbach’s alpha coefficient = 0.794

In this sample, vaccination coverage was 99.1%, with 28 participants reporting having received two doses, 197 reporting having received three doses, and 84 reporting having received four doses, respectively. Approximately 45% of the participants received the AstraZeneca vaccine as the first dose, and more than 80% of the participants responded that they were not afraid to be vaccinated against COVID-19. There was no significant difference in the distribution of men and women regarding the degree of fear of receiving the vaccine (p = 0.653). Among men, the majority declared that they did not have a preference for a type of vaccine (55.8%); among the most preferred were Pfizer (25.8%), followed by AstraZeneca (11%). Among the women, 58.9% said they did not have a preference for a type of vaccine, and the preferred vaccines were also Pfizer (24.1%) and AstraZeneca (9.5%).

In the online survey, 81 out of a total of 312 respondents reported having underage children. All of them confirmed vaccinating their children. Hence, a vaccination rate of 100% was observed. Among these 81 participants, 96% reported that vaccinating their child is important for the health of other people in the community and that vaccinating them is a good protective measure. However, 23.5% believed that the new vaccines against COVID-19 posed more risks than other vaccines (such as the flu vaccine), and 47% said they were concerned about adverse effects related to the vaccine against COVID-19.

Table 3 shows the groups (clusters) generated from the K-means analysis used to identify groups with similar characteristics within the university community. The analysis identified three groups, named according to the study variables that presented the greatest difference between the groups: ‘health workers’, ‘younger people’ and ‘people with chronic diseases’. The group with the highest score on the COVID-19 Fear Scale, which also had the highest number of health workers, had a mean age of 39 years. The group of younger participants had a mean age of 23 years and, therefore, had the lowest level of education, a lower frequency of chronic diseases and a lower degree of fear about being vaccinated against COVID-19. Finally, the third group, people with chronic diseases, included more men, with a higher mean age (55 years), higher education level, lower occurrence of SARS-CoV infection and lower COVID-19 Fear Scale score.

**Table 3:** Final cluster centers (centroids; means) of the sociodemographic and clinical variables: Online survey in the university community (n = 312) (Differences that identify the *cluster* are in bold).

	Cluster 1 “health workers”	Cluster 2 “younger people”	Cluster 3 “people with chronic diseases”	F test
Number of cases	99	161	52	
Sex	1	1	0	3.30
Age	39.02	23.43	**55.49**	1400.60
Education level	2.85	**2.13**	2.96	179.02
Health worker status	**0.15**	0.14	0.13	0.04
Chronic diseases	0.31	0.15	**0.37**	7.69
Already contracted COVID-19	**0.58**	0.50	0.37	3.05
Fear of COVID-19 (total score)	**14.23**	13.67	12.81	1.62
Degree of fear in being vaccinated against COVID-19	0.36	**0.18**	0.35	3.34

Convergence was achieved in 7 iterations.

### In-person survey

A total of 1896 individuals answered the questions face-to-face in health units: 515 men, 1365 women and 16 people who did not report their sex. The ages ranged between 18 and 93 years (mean 45 years). Table 4 presents the description of the sociodemographic and clinical variables of the participants assisted by the FHS.

**Table 4:** Sociodemographic and clinical description characteristics of participants from FHS: In-person survey.

		Women (n = 1365)	Men (n = 515)
**Age** [Mean (SD)]	Years	43.7 (16.0)	47.0 (18.6)
**Skin color** (%)	White	38.5	43.5
	Black	23.8	23.1
	Brown/Yellow	35.1	31.4
	Indigenous	0.1	0.2
	Not reported		
**Education level** (%)	Illiterate	0.1	0.2
	Incomplete elementary	31	31.1
	Complete elementary	4.3	5.5
	Incomplete high school	5.7*	11.7*
	Complete high school	35.1*	27.8*
	Incomplete graduation	8.7*	11.7*
	Graduated	8.3	7
	Graduate	6.7	4.9
**Family income** (%)	<0,5–1,5 wages/person	56.9*	49.3*
	1,5–3 wages/person	14.7	19.2
	3–6 wages/person	5	6.4
	>6 wages/person	0.9*	1.9*
	Not reported	22.5	23.1
**Occupation** (%)	Employed	45*	51.8*
	Unemployed	17.8*	9.9*
	Did not answer/other	37.2	38.3
**Health worker** (%)	yes	19.6*	7.7
**Chronic disease** (%)	yes	49.9	45.7

*p<0.05 (chi-square test)

Among the participants who visited basic health units, the majority (40%) reported that they were white, followed by brown/yellow (32%) and black (23%), and had completed high school (30%). There was a difference in the distribution of educational levels between males and females, with more females having completed high school and more males having not completed college. In terms of family income, most participants (approximately 50%) declared an income of up to 1.5 times the minimum wage per family member, and 50% declared having an occupation (formal or informal employment).

A higher frequency of participants reported having a chronic disease (50% of women and 46% of men) compared to participants from the university community, possibly due to the place of recruitment of individuals (basic health units).

Table 5 shows the descriptive analysis and comparison of the variables related to COVID-19 between sexes. The mean scores for the Fear of COVID-19 scale were 17.6 (±6.1) for women and 16.2 (±5.9) for men. A greater proportion of women responded that they had already contracted COVID-19 and were afraid of contracting it again (p<0.05). The perceived risk of contracting the disease was not different between the sexes.

**Table 5:** Description of participants from FHS according to variables related to COVID-19: In-person survey.

		Women (n = 1365)	Men (n = 515)
**Already contracted COVID-19** (%)	yes	40.5‡	33.3‡
**Family member/friend/acquaintance died of COVID-19**	yes	53.4	50.6
**Fear of contracting COVID-19**	yes	63.9 ‡	50.3 ‡
**Perceived risk of contracting COVID-19**	low	29.1	31.2
	moderate	40.8	40.7
	high	30	28.1
**Fear of being vaccinated against COVID-19** (%)	none	49.3 ‡	62.3 ‡
	low fear	23.2	20.7
	moderate fear	19.3	12.1
	very afraid	8.1 ‡	5 ‡
**COVID-19 Fear Scale†** [Mean (SD)]	I am more scared …	2.7* (1.2)	2.5* (1.2)
	I feel uncomfortable thinking …	2.8 (1.2)	2.7 (1.2)
	My hands are wet …	2.1* (1.0)	1.9* (0.9)
	I am afraid of losing my life …	3.2* (1.3)	2.9* (1.4)
	When watching the news … I become nervous or anxious	2.7* (1.2)	2.5* (1.2)
	I cannot sleep …	2.0* (1.0)	1.8* (0.9)
	My heart races or flutters …	2.2* (1.1)	2.0* (1.0)
	Total score	17.6* (6.1)	16.2* (5.9)

‡ p<0.05 (chi-square test)

*p<0.05 (ANCOVA test with adjustment for age)

† Cronbach’s alpha coefficient = 0.878

Regarding vaccination coverage, among the 1896 respondents, 1564 (∼83%) reported having been vaccinated, 314 (∼16%) did not respond, and 18 individuals (∼1%) reported not having been vaccinated. Of the total number of vaccinees, 30% received the AstraZeneca, 24% received the Pfizer and 23% received the CoronaVac vaccines as the first dose, and most participants (46%) reported no preference for a type of vaccine. Women reported a greater perception of fear of being vaccinated against COVID-19, in addition to scoring higher on the COVID-19 Fear Scale (p<0.05) (Table 5).

The ordinal logistic model adjusted to predict the degree of fear of being vaccinated against COVID-19 among users of basic health units showed a significant association with the independent variables sex (female) and score in the physiological domain of the COVID-19 Fear Scale (Table 6). The degree of fear of being vaccinated was 1.6 times higher among women than among men. Additionally, the degree of fear was 1.10 times higher for each additional point on the COVID-19 Fear Scale (physiological domain).

**Table 6:** Ordinal logistic model adjusted to predict the degree of fear of being vaccinated against COVID-19† among users of FHS (n = 1059).

Predictor variables	B	Wald’s chi-square	Sig	Exp (B)	CI (95%) Exp (B)
Age	-0.003	0.289	0.591	0.997	0.988–1.007
Sex (female)	0.454	9.892	**0.002**	**1.575**	1.187–2.090
Education level					
Incomplete elementary	0.098	0.006	0.938	1.103	0.096–12.734
Complete elementary	0.422	0.109	0.741	1.525	0.125–18.639
Incomplete high school	0.422	0.111	0.739	1.526	0.127–18.380
Complete high school	0.395	0.099	0.753	1.484	0.126–17.454
Incomplete graduation	-0.049	0.001	0.969	0.952	0.078–11.610
Graduated	0.241	0.036	0.849	1.273	0.106–15.329
Graduate	-0.035	0.001	0.978	0.965	0.079–11.730
Vaccinated	-1.433	3.794	0.051	0.239	0.056–1.009
Death due to COVID-19	0.070	0.320	0.572	1.072	0.842–1.364
History of COVID-19	0.016	0.017	0.897	1.016	0.794–1.301
Chronic disease	-0.142	1.011	0.315	0.868	0.658–1.144
Health worker	-0.073	0.174	0.677	0.929	0.658–1.312
Pregnant woman	0.139	0.078	0.780	1.150	0.432–3.062
Fear of COVID-19/emotional domain	0.028	1.795	0.180	1.028	0.987–1.071
Fear of COVID-19/physiological domain	0.095	10.026	**0.002**	**1.099**	1.037–1.165

†Question: ’How do you rate your degree of fear or insecurity about receiving the vaccine against COVID-19?’

Goodness of fit: Pearson’s chi-square (value/df) = 1.013; Omnibus test p<0.001.

Finally, two significant binomial logistic models were obtained to understand the motivations and fears regarding vaccination of underage children (Table 7). A total of 639 participants reported having dependent children under 18 years of age. Among them, 632 stated they had been vaccinated against COVID-19. Consequently, the vaccination rate among this demographic was 98.90%. For the question ‘Vaccinating my child is a good protective measure’, the variables sex, education level, health worker status and total score on the COVID-19 Fear Scale were included in the final model. An affirmative answer to the question was associated with male sex, having a lower education level and being a health worker (95% correct rate of the model). For the question ‘I am concerned that my child will develop an adverse event related to the COVID-19 vaccine’, the variables associated with an affirmative response were age and fear of COVID-19; that is, the fear of an adverse effect of the vaccine was associated with younger parental age and a greater fear of COVID-19 (percentage of correctness of the model, 72%).

**Table 7:** Binomial logistic models adjusted to predict the degree of fear of vaccinating children against COVID-19 among users of FHS (n = 425).

Question Model fit	Predictor variables	B	Wald	Sig	OR	CI (95%) OR
’Vaccinating my child is a good protective measure’	constant	4.361	11.390	<0.001	78.343	
Omnibus test p = 0,002	Sex (female)	-2.056	3.865	**0.049**	**0.128**	0.016–0.994
Nagelkerke’s R^2^ = 0,13	Education level	-0.300	4.878	**0.027**	**0.740**	0.567–0.967
Hosmer–Lemeshow test p = 0.895	Health worker	2.246	4.481	**0.034**	**9.455**	1.181–75.687
Overall percentage correct: 95.3%	Fear of COVID-19 total score	0.081	3.755	0.053	1.085	0.999–1.178
’I am concerned that my child will develop an adverse effect related to the COVID-19 vaccine’	constant	0.906	3.063			
Omnibus test p = 0,003	Age	-0.022	4.749	**0.029**	**0.978**	0.959–0.998
Nagelkerke’s R^2^ = 0,04	Fear of COVID-19 total score	0.051	7.249	**0.007**	**1.052**	1.014–1.092
Hosmer–Lemeshow test p = 0.680						
Overall percentage correct: 71.7%						

OR, odds ratio

The questions ‘Vaccinating my child is something important for the health of others in my community’ and ‘The new vaccines against COVID-19 have more risks than other vaccines (for example, the flu vaccine)’ did not generate significant models.

## Discussion

In the online survey conducted within the university community, female sex was associated with a greater perception of risk of contracting COVID-19 (p = 0.044) and with a higher score on the COVID-19 Fear Scale in relation to male sex (p = 0.022). Fear and anxiety are intrinsically related emotions [23]. Female sex has been associated with a greater fear of COVID-19 in several studies [19]. The particularities of the female hormonal physiology related to the menstrual cycle and reproductive stages are associated with the structures involved in the genesis of fear and anxiety. In this context, the hormonal fluctuations that occur during this period can alter the behavior of the hippocampus and the hypothalamus-pituitary-adrenal axis, which are the main structures related to the physiology of fear and anxiety [24]. In addition, it has been suggested that the greater routine burden of women with issues related to family and work are contributing factors to a higher prevalence of anxiety and fear among women [25, 26].

There was no significant difference in the fear of receiving the vaccine between men and women (p = 0.653). Although women reported a higher perceived risk of contracting the infection and a higher score on the Fear Scale, these variables did not reflect vaccine hesitancy. The fear of contracting the infection was only one of the factors related to the acceptance of immunization. However, the fact that this was a university population, associated with higher levels of income and education, may have generated greater confidence and acceptance of the vaccine [27–30].

In the analysis of the clusters generated, health workers had a higher total score on the COVID-19 Fear Scale. In general, these professionals tend to be closer to infected patients and have greater knowledge about the risks of the disease, which may reflect on the fear and anxiety of being infected [31]. The cluster of younger individuals had a lower degree of fear in being vaccinated against SARS-CoV-2. This result is controversial in the literature worldwide. Some studies report that younger people have greater vaccine hesitancy [14, 32, 33]. In part, this trend can be explained by the fact that this audience is more susceptible to receiving misleading information about vaccines and COVID-19 on social media [30]. In addition, some of these individuals believe they are less likely to contract severe forms of the disease, which may discourage vaccination [30, 33]. On the other hand, there are reports that demonstrate the absence of significant differences related to age [34] or even observed greater vaccine acceptance among younger individuals, which agrees with our results [30, 35]. This situation demonstrates that further clarification is still needed on the behavior of the younger public in Brazil regarding vaccines against COVID-19. Socioeconomic and political factors, given the troubled Brazilian political situation during the fight against the pandemic, may have differently influenced each age group with respect to vaccination, so that younger people were more likely to be immunized compared to other age groups, such as elderly individuals, contrary to what has been observed in many studies [35].

Finally, people with chronic diseases had a lower COVID-19 Fear Scale score. In this sense, although comorbidities are associated with more severe forms of the disease [36, 37], this fact did not generate greater fear of the disease among these individuals. This result is consistent with that in a previous study conducted in Brazil [19]. The group of patients with chronic diseases had a higher mean age. In general, older people tend to have less information and knowledge about the disease and its protective measures, which directly influences the fear of becoming infected [38]. The relationship between fear of COVID-19 and age shows divergent results in the literature [39–41].

In the population assisted by the FHS, women also reported a greater fear of COVID-19 according to the scale than men. As in the university population, such results were already expected, as discussed above. On the other hand, in this population, women had greater vaccine hesitancy against COVID-19 (p <0.05) than men in the same group, differing from women in the university community. This result corroborates the relationship between female sex and vaccine hesitancy already demonstrated in other studies [42–44]. However, a greater fear of COVID-19 was also associated with lower vaccine hesitancy in previous studies [30, 45]. Women tend to seek more health information (such as about the COVID-19 vaccine) and, consequently, may be exposed to more antivaccine content [46]. In summary, the present results demonstrate that fear of the vaccine possibly prevailed over fear of the disease and that other variables influenced vaccine hesitancy, such as lower income and education levels of participants in the in-person survey compared to those in the online survey.

Greater vaccine acceptance was associated not only with fear of contracting the infection but also with more years of schooling and higher socioeconomic levels. People with university degrees, for example, tend to be more likely to accept immunization when compared to people with elementary or secondary education. A lower income tends to be associated with a lower belief in the efficacy of vaccines [30, 42]. In addition, the present study was conducted in person, unlike a large portion of previously published studies on the subject, which used online data collection. This issue may have contributed to the fact that we had a more vulnerable audience, without access to computers and the internet, and consequently, less access to information [47]. The fact that women in the population assisted by the FHS (with lower income levels and fewer years of education) were more afraid of being vaccinated against COVID-19 than women in the online survey indicates that the most socioeconomically vulnerable population still lacks greater clarification on the importance and safety of vaccination to increase the desire for Immunization. A direct association has been reported between social vulnerability as a limiting factor in access to health and information [47].

Brazil has one of the largest immunization programs in the world, the PNI, which belongs to the SUS. Historically, the antivaccination movement has not had a major impact on the country. However, in recent years, this movement has intensified and contributed to the drop in vaccination coverage rates of several immunizing agents [5, 48]. In the context of COVID-19, the dissemination of fake news on social networks about possible harm caused by the vaccine, among other messages without scientific evidence, is notable [5, 49] and has substantially contributed to vaccine hesitancy and fear [5].

Regarding the vaccination of children, there are still few studies that have attempted to understand the feelings of parents regarding the vaccination of their children against COVID-19, especially in the Brazilian context. The present study showed that a positive answer to the question “Vaccinating my child is a good protective measure” was associated with lower parental education levels and a profession in the health area, as well as with male sex. A study conducted in Italy analyzed socioeconomic determinants of vaccine hesitancy and refusal and reported that a health profession did not influence the immunization of children. However, a low parental education level was associated with immunization refusal [50]. Other studies have reported that higher education levels lead to a greater interest in vaccinating children [27–29, 51].

In the present study, the answer “yes” to the question “I worry about my child developing an adverse effect related to the vaccine against COVID-19” was associated with a younger paternal age and greater fear of COVID-19. This may be associated with the minimal experience of parents regarding the vaccination of their children. Furthermore, according to Almuqbil et al., 2023 [52], young parents with low education levels are more greatly influenced by fake news or misinformation and, therefore, are hesitant to vaccinate their children.

The fear of adverse effects is an important variable associated with vaccine hesitancy and refusal [53]. In addition, this fear is largely related to the persistence of incorrect knowledge as a result of the spread of misinformation about vaccines [27]. Considering that much of the fake news regarding immunization is propagated over the internet [5], an environment in which there is a predominance of younger children, the result in our study that young fathers had a greater fear of side effects was consistent. However, it is worrisome that a considerable number of Brazilians report such concerns, as this indicates that communication with the public (vaccination campaigns and “days of action”, among others) has not been efficient in generating confidence in vaccines, which may compromise vaccination coverage. Trust is the main indicator capable of explaining vaccination behavior [54].

The present study has a cross-sectional design. Nevertheless, the inclusion of a large sample size and the innovative exploration of vaccine hesitancy in relation to university and primary health public system environments provides valuable insights for the development of public health policies. The results of the present study showed, in general, that women with greater socioeconomic vulnerability and lower education levels were more fearful of COVID-19 and more hesitant to receive vaccines. Additionally, parents with higher education levels were more likely to vaccinate their children, while younger parents were more fearful of adverse effects. Thus, investing in the communication and demystification of false beliefs related to vaccines is a fundamental measure to mitigate the fear of vaccines to increase immunization rates, whether among adults, children or adolescents. Vaccination is crucial for reducing Covid-19 mortality by preventing severe cases and hospitalizations. Prioritizing and promoting widespread vaccine uptake is essential in our collective responsibility to safeguard global well-being.

In conclusion, the research findings underscore the importance of implementing targeted communication strategies to disseminate accurate information about COVID-19 vaccines, addressing common misconceptions and emphasizing their significance in preventing disease spread. Additionally, ensuring easy access to vaccination services and providing resources for healthcare providers to engage effectively with patients is crucial. Moreover, community engagement initiatives led by local leaders and organizations play a pivotal role in promoting vaccine acceptance, dispelling misinformation, and monitoring vaccination attitudes and behaviors for informed decision-making. By adopting these strategies, we can minimize vaccine hesitancy and increase vaccination coverage.
